# The barriers to whole-grain consumption among Iranian students

**DOI:** 10.1038/s41598-022-19606-6

**Published:** 2022-09-08

**Authors:** Mohammad Ariya, Zahra Esmaeilinezhad, Mohammad Mehdi Naghizadeh, Mohammad Mehdi Dindarloo, Farzaneh Karimi, Fatemeh Kaveh, Sahar Marzban, Kimia Hormozi, Reza Barati-Boldaji

**Affiliations:** 1 Noncommunicable Diseases Research Center, Fasa University of Medical Sciences, Fasa, Iran; 2 Department of Nutrition, Fasa University of Medical Sciences, Fasa, Iran; 3 Nutrition Research Center, School of Nutrition and Food Sciences, Shiraz University of Medical Sciences, Shiraz, Iran; 4 Master Student of Biostatistics, School of Health, Mazandaran University of Medical Sciences, Mazandaran, Iran; 5 Shiraz Education Department, Shiraz, Iran; 6 Fasa Education Department, Fasa, Iran; 7 Gastroenterohepatology Research Center, Shiraz University of Medical Sciences, Shiraz, Iran

**Keywords:** Diseases, Health care

## Abstract

Studies show that regularly consuming whole grains reduce the risk of obesity and a wide range of chronic diseases. Despite this, studies reveal that students are consuming fewer whole grains. In the present study, we aimed to investigate the barriers to the consumption of whole grains among Iranian students. This cross-sectional study examined students at Fasa, Iran in 2020–2021. The online questionnaires were completed by students after receiving informed consent. Statistical analysis was performed using SPSS 26 and Chi-square, t-test, and logistic regression (*P* > 0.05). The current study involved 1890 students (1287 (68.1%) girls and 603 (31.9%) boys). Despite the preference for white flour bread among 53.8% of all students, 77.4% ate other whole-grain products, and 75.2% consumed all products at least once a week. Additionally, barriers such as access issues (70.5%), family supply issues (91.8%), lack appeal (72.8%), non-consumption by classmates (96.2%), and high prices in recent years (43.9%) were identified as obstacles to whole grain consumption. Furthermore, white bread eating students had significantly lower appetite levels and tended to eat fast food more often than those who ate whole grains (*P* < 0.05). We found that slightly more than half of the participants preferred to eat bread prepared with refined flour. Several other factors, including lack of access, lack of attractiveness, product price, parents not purchasing whole-grain products, students not paying attention to nutrition labels, peers’ effect, and eating with friends instead of family, also contribute to students avoiding whole-grain products.

## Introduction

According to American Association of Cereal Chemists (AACC), whole grains contain intact, ground, or cracked caryopsis, which is composed of the endosperm, germ, and bran^1^. Besides, processed products that use whole grains or part of them are also considered whole grains, provided that these products contain nearly the same amount of essential nutrients as the original grain seed^1^.

According to epidemiological studies, consuming whole grains regularly reduces the risk of non-communicable diseases such as cardiovascular disease, type 2 diabetes, some cancers, metabolic syndrome, and overall mortality^2–6^. Furthermore, these products can lead to improved weight status and reduced waist circumference^7^. Moreover, whole grains are a good source of fiber, vitamins, minerals, and other phytochemicals such as phenolic compounds, carotenoids, inulin, as well as other bioactive phytochemicals that can greatly prevent chronic non-communicable diseases^5^. Despite these benefits, adolescents have for a long time consumed fewer whole grains^8^.

According to a study, more than 40% of children and adolescents do not consume products that contain more than 51% whole grains^9^. The U.K.'s National Dietary Survey of British Adults (NDNS 2008e2011) also reported that 15% of children/adolescents did not consume any whole grain foods and their total average intake was only 13 g/day^10^. Furthermore, a study done in 1994–1996 to assess whole grain intake in children and adolescents found that in individuals 2 to 18 years of age, whole grain consumption was 0.9 servings per day^11^. Whereas according to Healthy People 2010, 6 servings of grain products per day (with at least 3 being whole grains) is necessary for good health^12^.

It is worth mentioning that there has been a remarkable change in Iranians' eating habits recently, and these changes are much more evident in the younger generation^13–15^. Several studies have reported that there are many reasons for this matter, including urbanization, social development, rapid demographic change, and the absence of significant economic improvement in Iran^13–16^. These issues influence nutrition transition and may reduce physical activity. As a result, the occurrence of obesity and being overweight could be unavoidable. Other studies have also shown that the dietary pattern of most Iranians gradually gets worse with the tendency to consume unhealthy foods such as fast foods^13,14^. These inappropriate dietary patterns among the Iranian population could be due to an unawareness of the importance of nutrients and micronutrients^14^. For instance, there is a report showing that the consumption of cereal and whole bread in Iranians is approximately 40% lower than that in other countries^17^. Therefore, it is crucial to evaluate the factors that cause the non-use of a healthy diet, such as whole grains, in Iran, especially among adolescents.

Considering the benefits and properties of whole grains, the Dietary Guidelines for Americans recommend eating at least 3 oz-equivalents of whole grains per day^18^. There should be at least two servings for children ages 4 to 8, and three servings for teenagers ages 9 to 18^19^. Nevertheless, studies show that children and adolescents consume less than one servings whole grain per day^11,20^. According to other studies, consumption of whole-grain products has decreased significantly around the world in recent years, and this has also been observed among students^9,20–23^. Hence, it seems that finding barriers to students' consumption of whole-grain products is of special importance.

On the other hand, whole grains are considered a rich source of fiber. It is recommended that children 4 to 8 years, girls 9 to 18 years, boys 9 to 13 years, and boys 14 to 18 years consume 18, 26, 31, and 38 g of fiber per day, which can be significantly obtained through consuming whole grains^19^. However, children and adolescents receive much less fiber and whole grains during the day^19^. Furthermore, as whole grains contain a variety of nutrients, such as n-3 fatty acids, sulfur-containing compounds such as methionine and cystine, oligosaccharides such as fructans, a variety of minerals and vitamins namely P, Mg, Ca, Na, K, B vitamins, flavonoids such as isoflavonoids, and other compounds such as betaine, choline, and melatonin, consumption of them is essential for the body and may prevent various diseases^1^. Therefore, students should get acquainted with this food pattern from beginning and include such foods in their diet.

Aside from the issues raised above, as obesity and overweight rates are rising and a significant percentage of Iranian students are at risk of being overweight and obese, they should be provided with basic nutrition advice and principles to prevent these issues^24^. As a result, epidemiological studies show that whole grains reduce markers of overweight and obesity, including body weight, BMI, waist circumference, and body fat percentage^25^. Furthermore, if eating whole grains is a habit that is formed from childhood or adolescence, it will have a significant impact on a person's health, food choices, and perhaps chronic diseases that he will struggle with in the future. Considering the above explanations and the importance of uncovering the barriers that prevent students from consuming whole grains^23^, the present study aimed to investigate the factors that influence whole grain consumption and the relationship between whole grain consumption and dietary pattern, appetite, and digestive problems. It is noteworthy to mention the present study is the first to investigate the barriers that prevent Iranian students from consuming whole grains.

## Results

Participating in the study were 1890 students, including 1287 girls (68.1%) and 603 boys (31.9%). Details of the demographic and anthropometric information of the study population are listed in Table 1. Table 2 shows the results of the whole grain barriers questionnaire. According to this table, more than half of all students (53.8%) consumed white-flour bread, and this percentage in both girls and boys was higher than that of students who preferred to eat whole-grain bread.

**Table 1:** Demographics and anthropometry information of the students.

	Mean	SD
Age (years)	12.1	3.1
Weight (kg)	47.99	19.62
Height (cm)	148.96	16.81
Waist circumference (cm)	71.84	13.82
Wrist circumference (cm)	14.92	2.01

	Frequency	Percent
**Gender**		
Girl	1287	68.1
Boy	603	31.9
**Level of education**		
Elementary	966	51.1
High school	924	48.9

**Table 2:** Information obtained from the whole grain barriers questionnaire.

			Total		Girl		Boy	
			N	%	N	%	N	%
Which type of bread would you choose to consume?		Processed bread or refined grain (Bread prepared with white flour)	1017	53.8%	690	53.6%	327	54.2%
		Whole meal bread (Bread prepared with whole grain flour)	873	46.2%	597	46.4%	276	45.8%
How do you feel about/what do you think of whole-grain foods?		They are very good	1102	58.3%	745	57.9%	357	59.2%
		They do not look good	121	6.4%	91	7.1%	30	5.0%
		They seem useless	144	7.6%	110	8.5%	34	5.6%
		I have no sense in eating whole grains since these foods are not available for me	523	27.7%	341	26.5%	182	30.2%
**Are there good things/health benefits in whole-grain foods?**								
They contain fiber and are good for our health		No	778	41.2%	551	42.8%	227	37.6%
		Yes	1112	58.8%	736	57.2%	376	62.4%
They supply source of energy for a longer time		No	1185	62.7%	808	62.8%	377	62.5%
		Yes	705	37.3%	479	37.2%	226	37.5%
They contain different vitamins and minerals		No	1148	60.7%	762	59.2%	386	64.0%
		Yes	742	39.3%	525	40.8%	217	36.0%
They can be useful to prevent high blood pressure (Hypertension)		No	1638	86.7%	1122	87.2%	516	85.6%
		Yes	252	13.3%	165	12.8%	87	14.4%
They can be useful in preventing cancers		No	1654	87.5%	1121	87.1%	533	88.4%
		Yes	236	12.5%	166	12.9%	70	11.6%
They can be useful in preventing type 2 diabetes		No	1508	79.8%	1044	81.1%	464	76.9%
		Yes	382	20.2%	243	18.9%	139	23.1%
They can be useful for keeping the heart healthy and preventing cardiovascular disease		No	1375	72.8%	935	72.6%	440	73.0%
		Yes	515	27.2%	352	27.4%	163	27.0%
Have you ever tried/eaten other whole-grain products?		Yes	1462	77.4%	1005	78.1%	457	75.8%
		No	428	22.6%	282	21.9%	146	24.2%
How often do you consume whole-grain foods (bread and other products)?		I do not consume at all	562	29.7%	380	29.5%	182	30.2%
		1–3 times a week	1040	55.0%	715	55.6%	325	53.9%
		More than 4 times a week	288	15.2%	192	14.9%	96	15.9%
**What do you think are the factors that affect/influence your whole grain consumption?**								
They are not available to us		Yes	1333	70.5%	941	73.1%	392	65.0%
		No	557	29.5%	346	26.9%	211	35.0%
Our parents do not provide it for us and there is not enough variety of these products in the school		Yes	1735	91.8%	1184	92.0%	551	91.4%
		No	155	8.2%	103	8.0%	52	8.6%
Our classmates do not like whole grains, and neither do I (Image among peers)		Yes	1819	96.2%	1232	95.7%	587	97.3%
		No	71	3.8%	55	4.3%	16	2.7%
They are not attractive/appealing for me to consume		Yes	1366	72.3%	890	69.2%	476	78.9%
		No	524	27.7%	397	30.8%	127	21.1%
Do you feel whole-grain foods are more expensive than refined grain foods?		Yes	725	38.4%	464	36.1%	261	43.3%
		No	217	11.5%	142	11.0%	75	12.4%
		I don't know	948	50.2%	681	52.9%	267	44.3%
Have whole-grain foods become more expensive in the last two years?		Yes	830	43.9%	524	40.7%	306	50.7%
		No	25	1.3%	16	1.2%	9	1.5%
		I don't know	1035	54.8%	747	58.0%	288	47.8%
Does eating whole grains cause digestive problems such as bloating?		Yes	153	8.1%	106	8.2%	47	7.8%
		No	799	42.3%	499	38.8%	300	49.8%
		I don't know	938	49.6%	682	53.0%	256	42.5%
Do you think media (especially TV) is important and does it affect what you eat? It can influence for introducing you to whole grains?		Yes	869	46.0%	560	43.5%	309	51.2%
		No	444	23.5%	312	24.2%	132	21.9%
		I don't know	577	30.5%	415	32.2%	162	26.9%
Do you pay attention to the labels of the foods when you what to buy them?		Yes	1119	59.2%	730	56.7%	389	64.5%
		No	771	40.8%	557	43.3%	214	35.5%
Do you think you will start eating or increase your whole grain consumption in the future?		Yes	1394	73.8%	914	71.0%	480	79.6%
		No	496	26.2%	373	29.0%	123	20.4%
If a whole-grain option was readily available for you at an eat-out would you choose it instead of refined grains?		Yes	1108	58.6%	756	58.7%	352	58.4%
		No	782	41.4%	531	41.3%	251	41.6%
If you ate more meals with your family instead of friends, do you think you would eat more whole-grain foods?		Yes	1445	76.5%	954	74.1%	491	81.4%
		No	445	23.5%	333	25.9%	112	18.6%
Would you choose whole-grain foods for their health benefits even if they are not that tasty?		Yes	1344	71.1%	903	70.2%	441	73.1%
		No	546	28.9%	384	29.8%	162	26.9%

Students also were asked about the benefits of eating whole grains. Although a higher percentage of all students (58.8%) considered whole grains as a source of fiber, it seems that most students did not have enough information on other benefits of whole-grain products such as a source of energy for a longer time (62.7%), supplier of vitamins and minerals (60.7%), and prevention of non-communicable diseases such as hypertension (86.7%), cancer (87.5%), type 2 diabetes (79.8%), and cardiovascular diseases (72.8%) (Table 2).

We also found that although a higher percentage of students preferred white bread, a significant percentage of students (77.4%) responded affirmative to the question, "Have you ever tried/eaten other whole-grain products?". We also revealed that 75.2% of all students reported consuming whole-grain products at least once or more per week. However, 29.7% of students did not consume whole-grain products at all (Table 2).

Table 2 also shows that the barriers to consuming whole grain, include not availability (70.5%), didn't provide by parents or not having enough variety (91.8%), and not appealing (72.8%). Furthermore, the majority (96.2%) claimed that since their classmates did not consume these products, they did not want to. The matters about the price of whole-grain products and the effect of television also are displayed in this table. Additionally, about 74% of students responded positively to the question of "Do you think you will start eating or increase your whole grain consumption in the future?" and about 59% said that if whole-grain products are available in abundance, they prefer to consume them rather than white-flour products. Moreover, 76.5% of students reported that they would be more likely to eat whole grains if they ate more meals with their family rather than with friends. Furthermore, many (71.1% of the respondents) indicated that even if whole-grain products are not tasty, they would consume them due to their health benefits (Table 2).

In Table 3, the type of bread and the number of times consumed whole-grain products did not differ significantly between girls and boys (*P* = 0.8 and *P* = 0.76, respectively). Furthermore, these differences were not statistically significant across educational levels (*P* = 0.79 and *P* = 0.98). There was no significant difference found between age and type of bread consumed or frequency of consumption of whole-grain products (*P* = 0.4 and *P* = 0.9).

**Table 3:** The relationship between the type of bread and frequency of consumption with age, gender, and level of education in the students.

	Type of bread				Frequency of consumption					
	Refined grain/white flour		Whole grain		I do not consume at all		1–3 times a week		4 times a week or more	
	N	%	N	%	N	%	N	%	N	%
**Gender**										
Girl	690	67.8%	597	68.4%	380	67.6%	715	68.8%	192	66.7%
Boy	327	32.2%	276	31.6%	182	32.4%	325	31.3%	96	33.3%
*p*-value	0.802				0.765					
**Level of education**										
Elementary	517	50.8%	449	51.4%	288	51.2%	530	51.0%	148	51.4%
High school	500	49.2%	424	48.6%	274	48.8%	510	49.0%	140	48.6%
*p*-value	0.796				0.989					

	Mean	SD	Mean	SD	Mean	SD	Mean	SD	Mean	SD
Age (years)	12.04	3.12	12.16	3.12	12.08	3.18	12.12	3.09	12.03	3.12
*p*-value	0.404				0.907					

This study found a relation between the type of bread consumed and the qualitative status of iron deficiency anemia. The relationship held even after adjusting the data (*P* < 0.05) (Table 4). White flour consumption also resulted in significantly higher rates of nausea and vomiting (OR 2,08, 95% CI 1.08–4), gastroesophageal reflux (OR 2, 95% CI 1.02–3.33), and heartburn (OR 1.52, 95% CI 1.11–2.07) than whole-bread consumption (*P* < 0.05) (Table 4). The relationship between these factors was held even after adjusting the data (*P* < 0.05).

**Table 4:** The relationship between the qualitative status of iron deficiency anemia, digestive problems, dietary pattern, and level of physical activity with the type of bread in the students.

			Type of bread				Univariate analysis				Adjusted by age, sex, education			
			Refined grain/white flour		Whole grain		*P*-value	OR	95% CI		*P*-value	OR	95% CI	
			Count	%	Count	%			L	U			L	U
BMI		Normal	608	73.2%	545	74.5%								
		Obese	223	26.8%	187	25.5%	0.563	1.07	0.85	1.34	0.563	1.07	0.85	1.34
**Qualitative status of iron deficiency anemia**														
Do your hands and feet constantly fall asleep?		No	572	56.2%	593	67.9%								
		Yes	445	43.8%	280	32.1%	< 0.001	1.65	1.37	1.99	< 0.001	1.71	1.41	2.07
Do you get tired of doing simple things? (For example, climbing stairs and doing housework)		No	563	55.4%	542	62.1%								
		Yes	454	44.6%	331	37.9%	0.003	1.32	1.10	1.59	0.002	1.35	1.12	1.62
Do you still feel tired and drowsy after enough sleep (Almost 8 h a day)?		No	634	62.3%	609	69.8%								
		Yes	383	37.7%	264	30.2%	0.001	1.39	1.15	1.69	< 0.001	1.44	1.18	1.76
**Which digestive problems do you have?**														
Constipation		No	905	89.0%	784	89.8%								
		Yes	112	11.0%	89	10.2%	0.565	1.09	0.81	1.46	0.519	0.91	0.68	1.22
Nausea and vomiting		No	986	97.0%	860	98.5%								
		Yes	31	3.0%	13	1.5%	0.028	2.08	1.08	4.00	0.026	0.48	0.25	0.91
Bloating		No	919	90.4%	803	92.0%								
		Yes	98	9.6%	70	8.0%	0.218	1.22	0.89	1.69	0.199	0.81	0.59	1.12
Diarrhea		No	1002	98.6%	861	98.6%								
		Yes	14	1.4%	12	1.4%	0.995	1.00	0.46	2.18	0.944	0.97	0.45	2.12
Gastric reflux		No	967	95.1%	851	97.5%								
		Yes	50	4.9%	22	2.5%	0.008	2.00	1.20	3.33	0.006	0.49	0.29	0.81
Heartburn		No	900	88.5%	804	92.1%								
		Yes	117	11.5%	69	7.9%	0.009	1.52	1.11	2.07	0.006	0.65	0.47	0.89
**Dietary pattern**														
Do you skip breakfast most of the time?		No	684	67.3%	646	74.0%								
		Yes	333	32.7%	227	26.0%	0.001	1.39	1.13	1.69	0.001	1.41	1.15	1.73
Do you skip other meals (lunch and dinner) most of the time?		No	654	64.3%	614	70.3%								
		Yes	363	35.7%	259	29.7%	0.005	1.32	1.08	1.60	0.003	1.34	1.10	1.63
Do you skip your snack most of the time?		No	405	39.8%	389	44.6%								
		Yes	612	60.2%	484	55.4%	0.038	1.22	1.01	1.46	0.034	1.22	1.02	1.47
**Level of physical activity**		Sedentary	718	70.6%	539	61.7%								
		Inactive	89	8.8%	97	11.1%	0.018	0.69	0.51	0.94	0.012	0.67	0.49	0.91
		Moderate activity	61	6.0%	66	7.6%	0.050	0.69	0.48	1.00	0.036	0.67	0.47	0.97
		Active/very active	149	14.7%	171	19.6%	0.001	0.65	0.51	0.84	< 0.001	0.64	0.49	0.82

BMI, Body mass index.

According to Table 4, those who ate white or refined bread significantly skipped meals and snacks more than those who consumed whole-grain bread (*P* < 0.05). In this regard, students who ate more white bread were 1.3 times more likely to skip breakfast (OR 1.39, 95% CI 1.13–1.69), and this was also true for skipping other meals and snacks, even after adjusting the data (*P* < 0.05). Lastly, we found that whole-grain bread consumers were significantly more active (after adjusting the data) than students who ate refined grains (*P* < 0.05). In addition, Supplementary Table 1 shows that the relationships observed above are also valid for the frequency of consumption of whole grains (apart from the variables related to the elimination of meals and snacks).

As can be seen in Table 5, white flour consumption is associated with a significantly lower appetite (based on CNAQ) and this variable remains significant after adjusting data (*P* < 0.001). VAS data also support this association (*P* < 0.001). In addition, our analysis showed that white flour consumers are significantly more likely to consume junk foods (*P* = 0.001), fast food (*P* = 0.025) and soft drinks (*P* = 0.002) (even after adjusting the data). Additionally, we found that whole-grain bread consumers spent significantly less time to fall asleep (*P* = 0.003). In addition, the frequency of consumption of whole grains was significantly related to appetite level (both VAS and CNAQ), (*P* = 0.005) and (*P* = 0.009) respectively) (Supplementary Table 2).

**Table 5:** The relationship between appetite level, consumption of fast food, and sleep quality with the type of bread in the students.

	Type of bread				Univariate	Adjusted by age, sex, education
	Refined grain/white flour		Whole grain		*P*-value *T*-test	*P*-value Linear regression
	Mean	SD	Mean	SD		
Appetite level (according to CNAQ)	29.4	4.48	30.34	4.38	< 0.001	< 0.001
Appetite level (according to VAS)	6.79	2.56	7.34	2.43	< 0.001	< 0.001
Consumption of junk food (chips, puffs, etc.) per week	2.2	1.87	1.93	1.74	0.001	0.001
Consumption of fast food per week	0.96	1.2	0.84	1.03	0.025	0.019
Drinking soda/soft drinks per week	1.57	1.75	1.34	1.51	0.002	0.002
The amount of sleep during 24 h a day (hour)	8.25	1.4	8.21	1.33	0.463	0.043
Length of time it took for the student to fall asleep (minute)	28.58	27.21	25.12	23.11	0.003	0.002
Sleep duration during the day (minute)	27.31	40.53	29.98	40.81	0.155	0.188

CNAQ, Council of Nutrition Appetite Questionnaire; VAS, visual analog scales.

## Discussion

Results of our study demonstrate that a higher percentage of students prefer white bread and that a significant portion of the participants was unaware of the benefits of eating whole grains. Furthermore, we discovered that about one-third of students did not consume any whole-grain products, and that approximately two-thirds did not find whole-grain products appealing or available.

The results of our study suggest that consumers may not consume whole grains for several reasons, including lack of availability, unattractiveness, and high price compared to products made with white flour. Other studies cite many factors as a poor whole-grain intake and a reluctance of students to consume whole grains. These factors include being a girl, lower household income, lack of awareness of the health benefits, household eating habits, difficulty identifying foods containing whole grains due to misleading labels, dislike of the taste, texture, and appearance of these foods, lack of availability, and their higher price than refined grains^7,21,26,27^. As children and adolescents dislike bitter foods, and whole-grain foods may be a little bitter, this may be one of the reasons why this group is hesitant to consume whole grains^28^. Due to its darker color as well as its coarser texture can also negatively impact school children's consumption of whole grains^28^. Another study suggests that one of the reasons students do not eat whole grains is that many households do not know how to cook whole-grain products^26^. All of these factors lead students to avoid products that contain whole grains, and therefore, consume products that contain refined grains.

In Iran, a study that directly examined the barriers to consume whole grains was not found based on our search. However, other research stated that many factors contribute to following an unhealthy diet pattern, especially among students. Among these factors, we can refer to the residence in populous and large cities such as Tehran; differences in social norms in recent years; less consumption of traditional dietary patterns that contain enough cereals and legumes, especially among the younger generation; more consumption of calorie-dense foods such as fast food and soft drinks; lower household income; low socioeconomic status; low level of education; smoking; low level of physical activity and having a sedentary lifestyle; less consumption of fruit, vegetables and fiber-rich foods; low level of parents' literacy and nutritional knowledge; consumption of white rice as a staple food and also white bread or refined grains as the main dietary component; high consumption of saturated oil; high salt intake; lack of appropriate food labeling; failure to raise public knowledge through media; high prevalence of eating disorder among adolescents; skipping breakfast as a mean meal for consuming whole grain; and type of food that friends and peers' choosing^13,14,29–34^.

Income plays a crucial role in the field. According to our study, about 39 percent of study participants reported that whole-grain products are more expensive than white flour, and about 44 percent reported that prices have risen recently. A study that evaluated the trend of whole-grain consumption among adolescents in the United States between 2005 and 2012 found that although consumption of whole grains has increased significantly in high-income households, this increase was not observed in low-income households^26^. Despite this, the increase in high-income households was still much lower than the recommended amount for health^26^. According to this study, low-income households may not take the risk of buying food whose taste is not compatible with their favorite^26^. Therefore, perhaps government support and the lowering of the price of whole-grain products can increase the desire of all categories, especially students, to consume these products.

We observed in the present study that one of the reasons for people not consuming whole grains is lack of knowledge about their benefits. School-aged children were unaware of the benefits of whole grains, such as providing energy for longer periods and supplying vitamins and minerals. Additionally, many did not aware of the fact that eating whole grains can prevent many non-communicable diseases. Other studies have shown contradictory results. Although some studies suggest that the reason for reduced whole-grain consumption is lack of knowledge about the benefits of these products^11^, one study states that many adolescents are aware of the benefits of whole-grain foods, regardless of which specific disease is associated with low consumption of whole grains (7). According to another study, whole-grain consumption can be increased by properly educating people about health benefits^35^. Therefore, students and their parents might be more likely to consume whole grains if they are aware of what whole grains can do.

Based on the findings of the present study, age, gender, and educational level were not associated with the type of bread consumed or the frequency of consuming whole-grain products. Previous studies have reported contradictory results. Similarly, it has been demonstrated in some studies that gender is not associated with whole grain consumption^27^. By contrast, according to a UK study, young people (under 18 years old) consume a very low level of whole grains, with an average of only 7 g per day^36^. In addition, approximately half of the participants in the 7-day evaluation did not consume any whole-grain breakfast cereals, but this ratio significantly increased with age^36^. The rate of non-consumption of whole grains increased with age, according to another study conducted in the United States^37^. These differences can be explained by the different populations studied.

Our study results showed that students who ate white bread were 1.3 times more likely to skip breakfast, other meals, and snacks than those who ate whole-grain bread. Breakfast is a very important meal, so its’ absence can contribute to academic failure^38^. In a UK study, 15- to 18-year-old girls reported the lowest intake of whole grains for breakfast, which might be the result of teens skipping breakfast^36^. In addition, the study found that the major sources of whole grains consumed by British teenagers were breakfast cereals and bread, although half of the students refused to eat breakfast cereals containing whole grains and three-quarters refused to eat wholemeal bread^36^. Similar results have been reported in the USA^11,37^. These studies may not be comparable, as they were conducted in different years and different regions, as well as varying methodologies and lengths of time. In addition to this, the definition of whole grains and the recommended daily amount vary from country to country. It could also be a contributing factor that the results of this study differ from those of other studies since consumption of ready-to-eat cereals among Iranians are not common for breakfast, as opposed to other countries where these products are the most common source of whole grains^7^.

Our research showed that school-aged children who ate more white bread reported significantly more digestive problems such as nausea and vomiting, reflux, and heartburn. Several studies identified that eating whole grains can improve digestive problems such as heartburn, bloating, and irritable bowel syndrome^39^. In this study, we also found that students who ate white bread had significantly fewer appetites but consumed more soda and fast food. In this regard, some studies have found that adolescents who consume whole grain have a lower tendency to consume fast food^21^. On the other hand, a meta-analysis study found that consumption of whole grains versus refined grains significantly affected subjective appetite and, consequently, consumption of whole grains reduced the risk of overweight and obesity^40^. It seems that more research is needed in this area.

Adolescents' consumption of whole grains is influenced by individual, family and environmental factors^41,42^. Accordingly, we observed that students who ate more meals with their families instead of eating with friends were more likely to consume whole-grain products. In addition, we noted that 96% of students feel hesitant to consume such products because their peers do not consume them. Similarly, a study found that the availability of whole-grain foods at home or at school can have a positive effect on adolescents' consumption of whole grains throughout the day^27^. According to this study, one of the best ways to use whole-grain products at home is to use products that require the least preparation and cooking time^27^. Based on another study, adolescents share a lot of eating patterns with their friends, and whole-grain consumption was found to be associated positively with close friendships^43^. Furthermore, a study found that adolescents are influenced by their families in their food choices, which can change their attitude toward healthy eating and whole grains^44^. It may be possible to increase the intake of whole grains among students by controlling these factors, which have a considerable impact on their consumption or non-consumption of whole grains.

According to our study, about 40% of the study population failed to pay attention to nutrition labels when purchasing foods. Another study shows that adolescents have difficulty identifying whole-grain products^7^. The problem with whole-grain products in this respect is that whole grain labels vary from country to country and also the minerals and fiber levels of such products vary due to growing and soil conditions so that consumers cannot identify such products correctly^7,9,19^. In addition, recommendations for the consumption of whole-grain products vary from region to region and country to country and also there are different definitions of the amount of whole-grain products required for a healthy diet^1,23,45^. Generally, a whole-grain food contains at least 8 g of whole grains per 30 g of product, according to the American Association of Cereal Chemists International. In this definition, 30 g is considered as a standard serving of whole-grain products^46^. In contrast, epidemiological studies have introduced the arbitrary value of 25% of cereal breakfast as whole-grain food, due to the consumption of whole grains and the risk of non-communicable diseases^9,47^. According to another FDA definition, a product is considered a whole grain if it contains at least 51% whole grain ingredient(s) (by weight) per reference^9^. As a result, disagreements over the definition of whole grains confuse the community and create uncertainty for producers of products containing whole grains. Consumers, on the other hand, did not specify which products they considered rich in whole grains^7^. Thus, it may be possible to reduce this confusion in society by defining a whole grain product in one way and encouraging all groups, especially students, to consume them.

Even though we did not find a significant relationship between BMI and consumption of whole grain bread in the present study, our findings showed that consumers of whole grain bread were significantly more active. This can be explained by the fact that a person who consumes whole-grain products maybe is more committed to a healthy lifestyle and therefore has a higher level of physical activity. While the results of a study conducted in United States were similar to ours and they showed that consumption of whole grains was not associated with BMI^20^, other studies suggest otherwise. In this regard, other investigations have shown that whole grains can significantly prevent overweight and obesity, especially in students^40,48^. A study also conducted in the United States determined that consumption of more than 3 servings of whole grains in children 6 to 12 years old was not associated with control of overweight and obesity in this age group, whereas consumption of 1.5 to 3 servings of whole grains per day was positively and significantly associated with weight measures. Additionally, the BMI z-score in this study was significantly lower in adolescents aged 13 to 18 years who consumed the most whole grains during the day^19^. In another study, adolescent boys who ate whole grains for breakfast had a significantly lower risk of obesity^49^. The explanation for these differences could be under-reporting, sociodemographic factors, or dietary patterns prevalent in the study population. Furthermore, the duration of the intervention varies from study to study, resulting in conflicting results.

According to our results, despite the fact that whole grain products were not tasty, a significant percentage of students were willing to eat them for health benefits. Meanwhile, we observed that about half of the students said they were familiar with whole grains because of television. Social media has been shown to encourage more students to consume whole grains in other studies^44^. Therefore, by raising awareness, it may be possible to increase the consumption of whole grains among adolescents. Other suggestions for increasing whole grain consumption among students include (1) consuming fast food with pieces of bread made from whole grains; (2) cheaper prices of products containing whole grains than refined grains; (3) TV advertising the consumption of whole grains; (4) improving the taste of whole-grain products using existing technologies; (5) free snacks for students that contain whole grains. Other studies in this area include suggestions such as advertising, increasing the sensory appeal of whole-grain products, abundant availability in schools and stores, formulation of new foods, snacks higher in fiber content, lowering prices, conducting educational campaigns and tailoring products to young people, and improving labeling to encourage more students to consume whole-grain products^7,23^.

Although this study examined a significant population, it had some limitations. First, the economic status of student households was not evaluated, and this should be considered in future research. Second, because of the nature of the study, a causal relationship could not be established. Another limitation of this study is that the data was collected online and through self-reporting, meaning that there is a possibility of reporting errors. Iron deficiency anemia was also evaluated qualitatively in the present study, which recommends future studies should use clinical tests. It is suggested that longitudinal studies be conducted in the future to determine what factors prevent students from consuming whole grains.

## Conclusion

This study examined barriers to the consumption of whole grains among Iranian students for the first time. Approximately half of the participants in our study preferred to consume bread made with refined flour. This could be explained by the fact that students are unaware of the benefits of using whole grains, as, a significant number of students were not aware that whole grains have many health benefits. According to the current study, several other barriers prevent students from consuming whole-grain products, including lack of access, lack of attractiveness, products price, parents not buying whole-grain products, don’t enough attention to nutrition labels by students, peers’ effect, and eating with friends instead of family. Furthermore, encouraging students to eat breakfast can also help them consume more whole grains, since breakfast is an important step for improving whole grain consumption. As a result, television and other social media can influence students to consume more whole-grain products. In conclusion, while we need more studies, particularly longitudinal or prospective studies, to better understand this issue, policymakers can use the results of this study and the proposed solutions to increase whole grain consumption among students. The chance of the burden of future chronic diseases in society may be reduced in part by adopting such an approach.

## Methods

This cross-sectional study was conducted from 2020 to 2021 among Iranian students covered by Fasa Education Department. Fasa is located in Fars Province and southwestern Iran. It has a population of about 250,000 according to the most recent census. This study is part of the plan to control overweight and obesity in students, and the contact numbers of students were provided to us by the Fasa Education Department. In this research, first, study participants were sent an informed consent form online. If they wished to participate in the study and filled out the informed consent, they were given the phone number and address of the project manager so that they could easily contact us if they had any questions. After receipt of online informed consent, demographic and anthropometric checklist, the 8-item Council of Nutrition Appetite Questionnaire (CNAQ)^50^, physical activity questionnaire, and a modified questionnaire on barriers to whole grain intake^7^ were completed online by students. It is worth mentioning that the barriers to whole-grain consumption questionnaire was based on the Kamar et al.^7^ questionnaire, but the students were asked to choose multiple-choice answers for easier answering.

A demographic checklist included factors such as age, sex, education level, height, weight, waist, and wrist circumference. Students were clearly explained how to measure anthropometric indices through a video submitted. Body mass index (BMI) was calculated by dividing weight (kg) by height squared (cm). The next questionnaire measures barriers to consuming whole grains by asking questions about the level of knowledge, attitude, desire to consume whole grains, and frequency of consumption. Also it included questions about what prevents students from consuming whole grains^7^. These questions are listed separately in Table 2.

In the current study, since bread is considered as staple food in Iran, the consumption of whole grains or white flour was evaluated by the consumption of type of bread. In the CNAQ, responses are scored based on a 5-point scale (A to E) (A = 1, B = 2, C = 3, D = 4, E = 5) and the final score evaluates the appetite. This questionnaire is scored on a scale from 8 (minimum) to 40 (maximum)^50^. It is noteworthy to mention that the psychometric properties of CNAQ was assessed in the USA which significantly correlated with the total score of the appetite hunger and sensory perception questionnaire (AHSP) and its sub-domain. CANQ also had Cronbach’s alpha equal to 0.72^50^. It should be noted that in addition to the appetite questionnaire, visual analog scales (VAS) were also evaluated in the present study. VAS is an anchor term that is recognized as a method for assessing changes in appetite over time^40^. This questionnaire is graded on a scale from zero to ten, with zero indicating no appetite and 10 indicating the highest degree of appetite. The physical activity questionnaire also uses 3 questions to calculate a person's exercise habits and level of physical activity. These questions assess the frequency, intensity, and duration of physical activity, and when these three items are multiplied together, the level of physical activity is obtained. The final score of this questionnaire can vary from less than 20 (sedentary) to more than 81 (very active)^51^. In the final analysis, we divided people into four groups: sedentary, low-activity (not good enough), moderately active (acceptable but could be better), and active or very active. This questionnaire has been also validated by previous studies^51,52^.

The students were also asked about their dietary patterns and how to eat fast food by way of an online form, as well as questions about gastrointestinal diseases. During this study, the qualitative status of iron deficiency anemia was assessed through the checklist. This checklist included three questions, and it was based on the most important symptoms of iron deficiency anemia. The answer to these questions was either yes or no. The questions are presented in Table 4. Moreover, the quality of sleep based on how much sleep was given during 24 h a day and the length of time it took for the student to fall asleep. The questionnaire was sent to each student via their smartphones as a link due to the prevalence of Coronavirus disease (COVID-19). Notably, for students who were not able to answer or fully understand the questions due to their young age (first to fourth grade), the information was provided by a first-degree relative who lived with the student or who have accurate information from the student's circumstances.

In the present study, the inclusion criteria included all students from all educational levels who wanted to complete the questionnaire online. Exclusion criteria included: unwillingness to participate in the study, special diets such as vegetarianism, extreme disability such that the student is unable to respond, psychotic mental illness, diseases leading to lack of recall (such as Alzheimer's disease), gastrointestinal diseases such as celiac disease, autoimmune diseases such as multiple sclerosis, and a variety of cancers. It should be noted that as this project was conducted through an online call, all students who participated in the project were studied. The consent form and questionnaires were sent online to about 25,000 students and those who were interested, filled out the questionnaire and participated. It is necessary to mention that the study protocol was in accordance with the Declaration of Helsinki guidelines and was approved by the Institutional Review Board (IRB) of the Fasa University of Medical Sciences (Code: IR.FUMS.REC.1400.158).

### Statistical analysis

Results were presented as mean and standard deviation. A Chi-square test was used to compare qualitative variables. Independent t-tests were used to compare quantitative variables. The effect of confounding variables was eliminated by multivariate logistic regression. This model reported significance and odds ratio along with a 95% confidence interval. Calculations were performed using SPSS version 26. Probabilities less than 0.05 were considered significant.

### Ethical approval and consent to participate

The study protocol was following the Helsinki Declaration and was confirmed by the Ethics Committee of Fasa University of Medical Sciences (Approval Code: IR.FUMS.REC.1400.158). The participants were informed about the research objectives and a consent form through online was obtained from the subjects before starting the survey.

## Supplementary Information

Supplementary Information.

## Data Availability

The datasets used and/or analyzed during the current study are available from the corresponding author on reasonable request to the corresponding author.
