# Supplementary material for: The barriers to whole-grain consumption among Iranian students
Source: Sci Rep. 2022 Sep 8;12:15224. doi: 10.1038/s41598-022-19606-6 (PMC9453731; doi:10.1038/s41598-022-19606-6)
Supplement: Supplementary file 1 — Supplementary Information. [file 41598_2022_19606_MOESM1_ESM.docx]

**Supplementary Table 1.** The relationship between the qualitative status of iron deficiency anemia, digestive problems, dietary pattern, and level of physical activity with the type of bread and frequency of consumption in the students

|  | | | | Type of bread | | | | | |  | | Frequency of consumption | | | | | |  |
| --- | --- | --- | --- | --- | --- | --- | --- | --- | --- | --- | --- | --- | --- | --- | --- | --- | --- | --- |
|  |  |  |  | Refined grain/ white flour | | Whole grain | | | | P-value | | I do not consume at all | | 1 to 3 times a week | | 4 times a week or more | | P-value |
|  |  |  |  | Count | % | Count | | % | |  |  | Count | % | Count | % | Count | % |  |
| BMI | | Normal | | 608 | 73.2% | | 545 | | 74.5% | |  | 336 | 72.3% | 645 | 75.3% | 172 | 71.4% |  |
|  |  | obese | | 223 | 26.8% | | 187 | | 25.5% | | 0.563 | 129 | 27.7% | 212 | 24.7% | 69 | 28.6% | 0.324 |
| Qualitative status of iron deficiency anemia | Do your hands and feet constantly fall asleep? | | No | 572 | 56.2% | | 593 | | 67.9% | |  | 300 | 53.4% | 669 | 64.3% | 196 | 68.1% |  |
|  |  |  | Yes | 445 | 43.8% | | 280 | | 32.1% | | **<0.001** | 262 | 46.6% | 371 | 35.7% | 92 | 31.9% | <0.001 |
|  | Do you get tired of doing simple things? (For example, climbing stairs and doing housework) | | No | 563 | 55.4% | | 542 | | 62.1% | |  | 297 | 52.8% | 631 | 60.7% | 177 | 61.5% |  |
|  |  |  | Yes | 454 | 44.6% | | 331 | | 37.9% | | 0.003 | 265 | 47.2% | 409 | 39.3% | 111 | 38.5% | 0.005 |
|  | Do you still feel tired and drowsy after enough sleep (Almost 8 hours a day)? | | No | 634 | 62.3% | | 609 | | 69.8% | |  | 339 | 60.3% | 703 | 67.6% | 201 | 69.8% |  |
|  |  |  | Yes | 383 | 37.7% | | 264 | | 30.2% | | 0.001 | 223 | 39.7% | 337 | 32.4% | 87 | 30.2% | 0.004 |
| Which digestive problems do you have? | Constipation | No | | 905 | 89.0% | | 784 | | 89.8% | |  | 488 | 86.8% | 933 | 89.7% | 268 | 93.1% |  |
|  |  | Yes | | 112 | 11.0% | | 89 | | 10.2% | | 0.565 | 74 | 13.2% | 107 | 10.3% | 20 | 6.9% | 0.018 |
|  | Nausea and vomiting | No | | 986 | 97.0% | | 860 | | 98.5% | |  | 544 | 96.8% | 1024 | 98.5% | 278 | 96.5% |  |
|  |  | Yes | | 31 | 3.0% | | 13 | | 1.5% | | 0.025 | 18 | 3.2% | 16 | 1.5% | 10 | 3.5% | 0.041 |
|  | Bloating | No | | 919 | 90.4% | | 803 | | 92.0% | |  | 510 | 90.7% | 949 | 91.3% | 263 | 91.3% |  |
|  |  | Yes | | 98 | 9.6% | | 70 | | 8.0% | | 0.218 | 52 | 9.3% | 91 | 8.8% | 25 | 8.7% | 0.936 |
|  | Diarrhea | No | | 1002 | 98.6% | | 861 | | 98.6% | |  | 552 | 98.2% | 1027 | 98.8% | 284 | 98.6% |  |
|  |  | Yes | | 14 | 1.4% | | 12 | | 1.4% | | 0.995 | 10 | 1.8% | 12 | 1.2% | 4 | 1.4% | 0.592 |
|  | Gastric reflux | No | | 967 | 95.1% | | 851 | | 97.5% | |  | 529 | 94.1% | 1013 | 97.4% | 276 | 95.8% |  |
|  |  | Yes | | 50 | 4.9% | | 22 | | 2.5% | | 0.007 | 33 | 5.9% | 27 | 2.6% | 12 | 4.2% | 0.005 |
|  | Heartburn | No | | 900 | 88.5% | | 804 | | 92.1% | |  | 494 | 87.9% | 947 | 91.1% | 263 | 91.3% |  |
|  |  | Yes | | 117 | 11.5% | | 69 | | 7.9% | | 0.009 | 68 | 12.1% | 93 | 8.9% | 25 | 8.7% | 0.100 |
| Dietary pattern | Do you skip breakfast most of the time? | No | | 684 | 67.3% | | 646 | | 74.0% | |  | 379 | 67.4% | 741 | 71.3% | 210 | 72.9% |  |
|  |  | Yes | | 333 | 32.7% | | 227 | | 26.0% | | 0.001 | 183 | 32.6% | 299 | 28.7% | 78 | 27.1% | 0.165 |
|  | Do you skip other meals (lunch and dinner) most of the time? | No | | 654 | 64.3% | | 614 | | 70.3% | |  | 359 | 63.9% | 709 | 68.2% | 200 | 69.4% |  |
|  |  | Yes | | 363 | 35.7% | | 259 | | 29.7% | | 0.005 | 203 | 36.1% | 331 | 31.8% | 88 | 30.6% | 0.142 |
|  | Do you skip your snack most of the time? | No | | 405 | 39.8% | | 389 | | 44.6% | |  | 218 | 38.8% | 434 | 41.7% | 142 | 49.3% |  |
|  |  | Yes | | 612 | 60.2% | | 484 | | 55.4% | | 0.038 | 344 | 61.2% | 606 | 58.3% | 146 | 50.7% | 0.013 |
| Level of physical activity | | Sedentary | | 718 | 70.6% | | 539 | | 61.7% | |  | 427 | 76.0% | 655 | 63.0% | 175 | 60.8% |  |
|  |  | inactive | | 89 | 8.8% | | 97 | | 11.1% | | 0.001 | 38 | 6.8% | 126 | 12.1% | 22 | 7.6% | <0.001 |
|  |  | Moderate activity | | 61 | 6.0% | | 66 | | 7.6% | |  | 25 | 4.4% | 81 | 7.8% | 21 | 7.3% |  |
|  |  | Active/ Very active | | 149 | 14.7% | | 171 | | 19.6% | |  | 72 | 12.8% | 178 | 17.1% | 70 | 24.3% |  |

BMI; Body mass index.

**Supplementary Table 2.** The relationship between appetite level, consumption of fast food, and sleep quality with the type of bread and frequency of consumption in the students

|  | Type of bread | | | | Frequency of consumption | | | | | |
| --- | --- | --- | --- | --- | --- | --- | --- | --- | --- | --- |
|  | Refined grain/ white flour | | Whole grain | | I do not consume at all | | 1 to 3 times a week | | 4 times a week or more | |
|  | Mean | SD | Mean | SD | Mean | SD | Mean | SD | Mean | SD |
| Age (years) | 12.04 | 3.12 | 12.16 | 3.12 | 12.08 | 3.18 | 12.12 | 3.09 | 12.03 | 3.12 |
| p-value | 0.404 |  |  |  | 0.907 |  |  |  |  |  |
| Appetite level (according to CNAQ) | 29.4 | 4.48 | 30.34 | 4.38 | 29.51 | 4.67 | 29.8 | 4.4 | 30.56 | 4.17 |
| p-value | <0.001 |  |  |  | 0.005 |  |  |  |  |  |
| Appetite level (according to VAS) | 6.79 | 2.56 | 7.34 | 2.43 | 6.8 | 2.7 | 7.09 | 2.42 | 7.33 | 2.45 |
| p-value | <0.001 |  |  |  | 0.009 |  |  |  |  |  |
| consumption junk food (chips, puffs, etc.) per week | 2.2 | 1.87 | 1.93 | 1.74 | 2.21 | 1.9 | 2.01 | 1.72 | 2.04 | 1.97 |
| p-value | 0.001 |  |  |  | 0.102 |  |  |  |  |  |
| consumption fast food per week | 0.96 | 1.2 | 0.84 | 1.03 | 0.92 | 1.18 | 0.88 | 1.04 | 0.95 | 1.28 |
| p-value | 0.025 |  |  |  | 0.62 |  |  |  |  |  |
| Drinking soda/ soft drinks per week | 1.57 | 1.75 | 1.34 | 1.51 | 1.54 | 1.77 | 1.43 | 1.56 | 1.41 | 1.71 |
| p-value | 0.002 |  |  |  | 0.377 |  |  |  |  |  |
| The amount of sleep during 24 hours a day (hour) | 8.25 | 1.4 | 8.21 | 1.33 | 8.25 | 1.45 | 8.25 | 1.32 | 8.14 | 1.39 |
| p-value | 0.463 |  |  |  | 0.458 |  |  |  |  |  |
| The time it takes to go to deep sleep (minute) | 28.58 | 27.21 | 25.12 | 23.11 | 27.93 | 26.31 | 26.41 | 24.51 | 27.22 | 27.05 |
| p-value | 0.003 |  |  |  | 0.516 |  |  |  |  |  |
| Sleep duration during the day (minute) | 27.31 | 40.53 | 29.98 | 40.81 | 27.97 | 41.34 | 28.9 | 40.52 | 28.39 | 40.02 |
| p-value | 0.155 |  |  |  | 0.907 |  |  |  |  |  |

CNAQ; Council of Nutrition Appetite Questionnaire, VAS; visual analog scales.
